# Association between *Schistosoma mansoni* infection and fecal occult blood in schoolchildren in Mbita, Suba North sub-county, western Kenya

**DOI:** 10.1186/s41182-025-00814-5

**Published:** 2026-01-26

**Authors:** Joy C. Biegon, John Gachohi, Benard C. Ngetich, Sammy M. Njenga, Shinjiro Hamano, Evans Asena Chadeka

**Affiliations:** 1 Graduate School of Health Sciences, Kenya Medical Research Institute (KEMRI), Nairobi, Kenya; 2 Department of Environmental Health and Disease Control, School of Public Health, Jomo Kenyatta University of Agriculture and Technology (JKUAT), Juja, Kenya; 3 Institute of Tropical Medicine (NUITM), Nagasaki University, Kenya Medical Research Institute (KEMRI) Project, Nairobi, Kenya; 4 Eastern and Southern Africa Centre of International Parasite Control (ESACIPAC), KEMRI, Nairobi, Kenya; 5 Department of Parasitology, Institute of Tropical Medicine (NEKKEN), Nagasaki University, Nagasaki, Japan; 6 Washington State University Global Health Program, Washington State University, P.O BOX 72938, 00200 Nairobi, Kenya; 7 Paul G, Allen School of Global Health, Washington State University, WA99164, Pullman, USA

**Keywords:** *Schistosoma mansoni*, Fecal occult blood, Preschool-aged children, School-aged children, Intestinal morbidity

## Abstract

**Background:**

*Schistosoma mansoni* infection is highly prevalent in sub-Saharan Africa and is associated with significant intestinal morbidity in children. Current monitoring tools primarily assess infection status and intensity, which may underestimate the disease burden. Fecal occult blood (FOB) is a reliable indicator of bowel morbidity; however, its utility in intestinal schistosomiasis remains inadequately characterized. This study aimed to evaluate FOB as a surrogate marker of *S. mansoni*-induced intestinal morbidity among children in endemic areas of Kenya.

**Methods:**

A pre–post intervention study was conducted among preschool-aged (3–5 years) and school-aged (9–14 years) children in the Mbita Health Demographic Surveillance System along the shores and islands of Lake Victoria, Suba North sub-county, western Kenya. A total of 611 children from 10 primary schools were screened for *S. mansoni* infection before praziquantel treatment, and 584 were re-evaluated 6 weeks post-treatment. In addition to parasitological examination for *S. mansoni*, FOB testing, malaria diagnosis, point-of-care hemoglobin measurement, and soil-transmitted helminth assessments were performed both before and after treatment. Associations between *S. mansoni* infection and FOB positivity were analyzed using Pearson’s Chi-square test and logistic regression.

**Results:**

*S. mansoni* infection prevalence was high before treatment, affecting 66.5% of preschool-aged and 77.4% of school-aged children. Among *S. mansoni*-infected children, more than three-quarters tested positive for FOB. Six weeks after praziquantel treatment, the prevalence of both *S. mansoni* infection and FOB positivity declined significantly (infection: 19–21%; FOB: 25–29%; *P* < 0.01). Before treatment, preschool-aged children residing on islands had twice the odds of FOB positivity compared to those on the mainland (AOR = 2.0; 95% CI 1.2–3.4; *P* = 0.01), although this association was no longer evident post-treatment.

**Conclusions:**

Our findings demonstrate a significant association between *S. mansoni* infection and FOB positivity. These results suggest that FOB testing could be a useful indicator for monitoring treatment-associated reductions in intestinal morbidity due to *S. mansoni* in endemic settings.

## Introduction

Schistosomiasis, a prevalent neglected tropical disease, remains a significant global health concern. It affects over 230 million people worldwide, with an estimated 779 million at risk of infection. Predominantly in Africa, Asia, the Caribbean, and South America [1], schistosomiasis imposes a substantial burden, particularly in sub-Saharan Africa [2]. In this region alone, it accounts for up to 90% of infections, leading to an estimated loss of 3 million Disability Adjusted Life Years (DALYs) [3] and 200,000 related deaths annually [4]. The impact is most profound in impoverished populations [5].

The primary aetiological agent for schistosomiasis in sub-Saharan Africa is *Schistosoma mansoni*, transmitted by freshwater *Biomphalaria* spp. snails. It manifests as intestinal schistosomiasis, characterized by symptoms such as abdominal pain, diarrhea, blood in stool, polyposis, and rectal bleeding [6]. In Egypt, colorectal polyps are considered a potential cause of rectal bleeding in children that is regularly overlooked [7]. Anemia and hepatosplenic complications have also been associated with *S. mansoni* infection among schoolchildren in western Kenya [8, 9], underscoring the urgency of effective control measures.

Morbidity control has been the cornerstone of schistosomiasis management strategies, with mass chemotherapy employing praziquantel as the primary intervention. Targeting endemic communities, particularly school-aged children with the highest infection burden, has been pivotal [1]. More recently, efforts have expanded to include preschool-aged children, recognizing their vulnerability to infection [10]. Following years of mass chemotherapy, the prevalence of *S. mansoni* infection and intensity has reduced by over 50% [11–13], accompanied by a significant decline in schistosomiasis-associated morbidities [14, 15]. Nevertheless, morbidity reduction reported in these studies is related to reduced infection intensity, a proxy indicator that is likely not sufficient to measure the success of mass chemotherapy. Therefore, there is a pressing need for more precise indicators that gauge the effectiveness of schistosomiasis control programs.

In the context of *S. mansoni* infection, more research has focused largely on ultrasonographic examination of the abdomen and liver [14–16]. Evidence on the impact of treatment on intestinal morbidity in young children, however, remains scarce. For many years, the fecal occult blood (FOB) test, indicative of hidden blood in the stool, has been used as a screening test for intestinal morbidities, including those related to colorectal cancer [17, 18]. The importance of FOB as a potential surrogate marker of bowel morbidity linked to gastrointestinal parasites is also increasing [19–22]. While some studies have found evidence of a correlation between *S. mansoni* infection and FOB [23, 24], these findings have not comprehensively addressed the peak age group for schistosome infections, particularly children aged 9–14 years [6]. The chances of observing a reduction in morbidity after treatment are probably high in this age group. In this study, we sought to address this gap in knowledge by assessing the applicability of fecal occult blood as a surrogate indicator of *S. mansoni*-induced bowel morbidity in school-aged children (9–14 years) and preschool-aged children (3–5 years) as a comparator group. This study aims to bridge this gap by evaluating the utility of FOB as an indicator of *S. mansoni*-induced bowel morbidity in school-aged children (9–14 years) compared to preschool-aged children (3–5 years). By doing so, we seek to provide valuable insights into the effectiveness of current control strategies and contribute to the refinement of monitoring and evaluation efforts in schistosomiasis management.

## Methods

### Study area

**Fig. 1:**
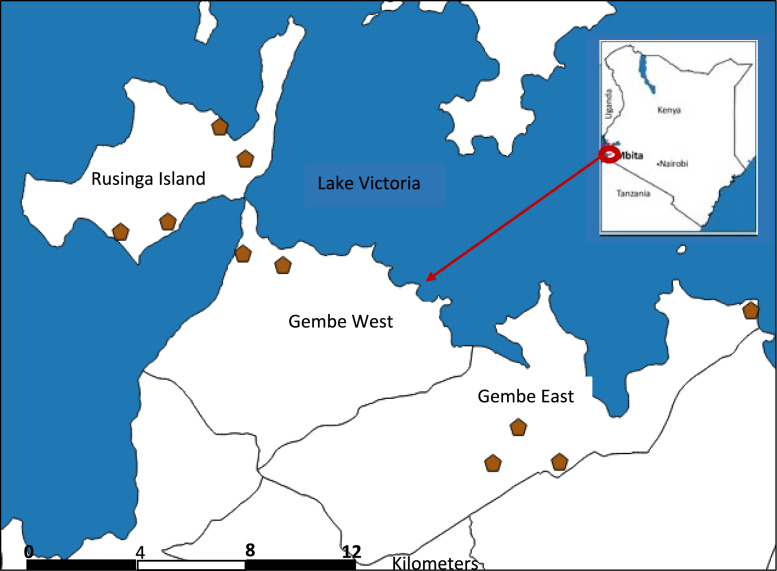
Map of Mbita HDSS showing the selected 10 primary schools

We conducted this study in Suba North sub-county, Homa Bay County, situated along the shores of Lake Victoria in western Kenya. We purposively selected four administrative locations: Gembe East and West on the mainland, and Rusinga East and West on the island, because they are part of Mbita Health and Demographic Surveillance System (HDSS). This system supports long-term follow-up and provides reliable geographical and population data (Fig. 1). The Mbita HDSS area is predominantly rural, characterized by fishing as the primary economic activity, and supplemented by small-scale farming, particularly among lakeside communities [25]. These locations are also known to have a high prevalence of *Schistosoma mansoni* infection, particularly among school-aged children, due to their proximity to Lake Victoria and the population’s frequent contact with lake water for domestic and recreational activities [26–29]. Owing to its endemicity for *S. mansoni* infection, the study area has been part of Kenya’s National School-Based Deworming Programme since it was initiated in 2012 to provide annual praziquantel (PZQ) treatment [13]. However, according to the records obtained from the sub-county Health and Education offices in Mbita, no mass drug administration (MDA) with PZQ had been conducted a year before the study; instead, only albendazole was administered during that period, targeting soil-transmitted helminths**.** This underscores both the epidemiological significance of the region and its relevance for evaluating the impact of control interventions.

### Study design

This study employed a pre–post design targeting preschool-aged children (PSAC) and school-aged children (SAC) in Suba North sub-county. We used cluster sampling to select ten primary schools from 54 primary schools in the four locations (Gembe East, Gembe West, Rusinga East, Rusinga West) covered by the Mbita HDSS. In each selected school, all pupils in pre-primary one (PP1) and fourth grade whose parents or legal guardians consented to the study were recruited. In Kenya, children typically enroll in grade 1 at 6 years, thus the majority of children in the PP1 class and 4th grade fall within the targeted age groups of 3–5 years and 9–14 years, respectively. This study recruited a total of 672 PSAC and SAC from a target population (PP1 class and fourth grade) of 3670 pupils in schools covered by the Mbita HDSS. We excluded pupils enrolled in other schistosomiasis-related studies. Trained field assistants from the local community obtained informed consent from parents or legal guardians at the household level and collected pupils’ details, including name, class, age, gender, and house geographical location.

### Parasitological examination

All enrolled pupils were provided with labeled specimen containers for stool and urine samples, each assigned a unique identification number, and were instructed on proper use. Trained field workers and class teachers assisted PP1 pupils with sample collection. During baseline, sample collection was conducted early in the morning over two consecutive days within the school premises, and all specimens were transported in cooler boxes with ice packs to the NUITM-KEMRI field laboratory in Mbita for analysis. Six weeks after praziquantel treatment, the field team returned to the schools to collect fresh stool and urine samples from the same participants for post-treatment assessment, following similar procedures. Follow-up visits were conducted during both the baseline and post-treatment phases to ensure comprehensive sample collection at each time point.

The Kato–Katz technique was used to detect *S. mansoni* and other soil-transmitted helminth infections, including hookworm, *Trichuris trichiura*, and *Ascaris lumbricoides*. Duplicate thick fecal smears were prepared per sample and examined by four laboratory technicians under a light microscope within 24 h of collection. Parasite egg counts per gram of feces (EPG) were determined following WHO guidelines, categorizing *S. mansoni* infection intensity as light (1–99 EPG), moderate (100–399 EPG), or heavy (≥ 400 EPG). Presence of *S. mansoni* antigen was also detected using a urine-based immunochromatographic circulating cathodic antigen (CCA) cassette test (Schisto POC-CCA™, Pretoria, South Africa), which provides qualitative results indicating either the presence (positive) or absence (negative) of the antigen. Positive infection status was defined by the detection of at least one parasite egg via the Kato–Katz method or a positive result on the CCA test, indicated by the presence of a pink band in the test area. These procedures were repeated 6 weeks after treatment for all study participants. Due to the high malaria transmission in the study area, *Plasmodium falciparum* infection was also assessed using an antigen-based rapid diagnostic test (STANDARD™ Q Malaria p.f Ag test, South Korea).

### Morbidity assessment: fecal occult blood (FOB) and anemia

Following the manufacturer’s instructions, FOB was detected using a rapid visual immunoassay (Polymed Accurate, India). The assay provided qualitative results (positive/negative) and cost approximately USD 1.5 per test at the time of the study. Stool specimens were collected using an applicator stick inserted into three different sites of the sample and then mixed vigorously with the extraction buffer. Three drops of the resulting solution were added to the test device well, and the results were read after 5 min. Any visible color change in the test region was recorded as positive. The FOB test was performed on stool samples collected from each participant both before praziquantel treatment and at follow-up.

We collected finger-prick blood samples from each study participant to assess hemoglobin levels using a Hemocue Hb 201^+^ photometer (HemoCue, Angelholm, Sweden). Previously, studies have reported an association between *S. mansoni* infection and anemia [8, 30]. Anemia was defined using age-specific hemoglobin thresholds: < 110 g/L for children under 5 years, < 115 g/L for those aged 5–11 years, and < 120 g/L for those aged 12–14 years. The severity of anemia was further classified based on age-specific cut-off values established by the World Health Organization [31].

### Treatment

All the study participants who tested positive for *S. mansoni* infection, confirmed by either Kato–Katz or CCA, were promptly administered a two-dose regimen of praziquantel treatment. The initial dose, calculated at 40 mg/kg body weight, was administered at baseline, followed by a second dose 2 weeks after the initial treatment, administered by licensed clinicians. Subsequently, all participants underwent re-examination 6 weeks after treatment, and those still found infected with *S. mansoni* were administered an additional single dose of praziquantel. Concurrently, pupils diagnosed with STH infections received albendazole, while those with malaria were treated with artemether–lumefantrine. Furthermore, pupils identified with low hemoglobin levels were supplemented with folate tablets to address any underlying deficiencies.

### Data analysis

Data collected during the study were entered into a Microsoft Excel spreadsheet and subsequently imported into R software version 3.6.1 for analysis. The normality of the data was assessed using the Shapiro–Wilk test. At the univariate level, continuous variables were summarized using the arithmetic mean, while categorical variables were presented as percentages. For the evaluation of *S. mansoni* intensity, EPG was computed by multiplying the number of schistosome eggs per Kato–Katz slide by 24, and then classified according to the established WHO guidelines into light (1–99 EPG), moderate (100–399 EPG), and heavy (≥400 EPG) infection categories. The Pearson Chi-squared test examined associations between categorical variables at the bivariate level. Subsequently, multivariate analyses were conducted employing logistic regression models to assess the association between *S. mansoni* infection and fecal occult blood while adjusting for potential confounders such as gender, geographical location, anemia, and malaria status. Statistical significance was determined at a *p* ≤ 0.05 with a 95% confidence interval (CI), indicating evidence of associations.

### Ethical consideration

This study received approval from the Institutional Scientific and Ethics Review Committees of the University of Eastern Africa, Baraton (UEAB/ISERC/39/07/2022) and the Kenya Medical Research Institute (KEMRI/SERU No. 4174), and further received a research license granted by the National Commission for Science, Technology, and Innovation (NACOSTI/P/22/19457), Kenya. Authorization to carry out the study was also obtained from both the Ministry of Education (MOE) and Health (MOH), Homa Bay County, Kenya. Before enrollment, written consent was obtained from parents or guardians, accompanied by assent from the participating children, ensuring full compliance with ethical standards and the protection of participants’ rights.

## Results

Out of the 672 enrolled participants, 611 were included in the analysis before treatment, with 584 re-examined 6 weeks after treatment. Among the 611 participants with complete data, 388 (63.5%) resided in mainland locations, specifically Gembe East and Gembe West. The majority of participants (52.2%) were preschool-aged children (PSAC), aged between 3 and 5 years, with a mean age of 4 ± 1 years. In contrast, school-aged children (SAC) had a mean age of 11 ± 1.3 years, with ages ranging from 9 to 14 years. Across both age groups (PSAC and SAC), the number of male participants was slightly higher than that of females. Demographic characteristics of the study population were comparable before and after treatment, as detailed in Table 1.

**Table 1:** Demographic profile of the study population

Variable	Number (%)		*P*-value (Chi^2^)
	Before treatment (*n* = 611)	After treatment (*n* = 584)	
Preschool-aged children (PSAC)	*n* = 319	*n* = 298	0.726
^a^Age (years)			
Range	3–5	3–5	–
Mean	4 ± 1	4 ± 1	–
Gender			
Male	163 (51.1)	151 (50.7)	0.979
Female	156 (48.9)	147 (49.3)	
Location			
Gembe East (Mainland)	123 (38.6)	117 (39.3)	0.978
Gembe West (Mainland	75 (23.5)	68 (22.8)	
Rusinga East (Island)	49 (15.3)	43 (14.4)	
Rusinga West (Island)	72 (22.6)	40 (23.5)	
School-aged children (SAC)	*n* = 292	*n* = 286	0.726
^a^Age (years)			
Range	9–14	9–14	–
Mean	11 ± 1.3	11 ± 1.3	–
Gender			
Male	157 (53.8)	154 (53.8)	1.000
Female	135 (46.2)	132 (46.2)	
Location			
Gembe East (Mainland)	93 (31.8)	93 (32.5)	0.999
Gembe West (Mainland	97 (33.2)	94 (32.9)	
Rusinga East (Island)	37 (12.7)	36 (12.6)	
Rusinga West (Island)	65 (22.3)	63 (22.0)	

^a^Age expressed as arithmetic mean ± SD (standard deviation)

Chi^2^: Pearson’s Chi-squared test

The prevalence of *S. mansoni* infection, fecal occult blood (FOB), anemia, malaria, and soil-transmitted helminths (STH) at two time points (before and 6 weeks after treatment) are presented in Table 2. Overall, the prevalence of *S. mansoni* infection, determined by both Kato–Katz and circulating cathodic antigen (CCA) tests, was 66.5% (95% CI 60.9–71.6) and 77.4% (95% CI 72.1–82.0) among PSAC and SAC, respectively. The majority of SAC with *S. mansoni* infection exhibited moderate (23.6%) and heavy (24%) infection intensities. Conversely, among infected PSAC, light infections (20.7%) were more common compared to moderate (10.7%) and heavy (7.8%) infections. Significant reductions (*P* < 0.001) in both the prevalence and intensity of *S. mansoni* infection were observed between the two time points for both PSAC and SAC. Before treatment, the prevalence of FOB was higher in SAC (66.1%) compared to PSAC (41.7%). According to WHO age-specific hemoglobin cut-off levels, anemia prevalence decreased among PSAC, from 48.0% (95% CI 42.3–53.6) to 38.6% (95% CI 33.1–44.4) (*P* = 0.023), while no significant change was observed among SAC (34.2–32.9%; *P* = 0.793). The prevalence of *Trichuris trichiura*, hookworm, and *Plasmodium falciparum* remained low throughout the study period, with no statistically significant differences before and after treatment. No cases of *Ascaris lumbricoides* were detected at either time point, as shown in Table 2.

**Table 2:** Prevalence of parasitic infections, fecal occult blood, and anemia before and after praziquantel treatment

Variable	Prevalence, % (95% Cl)		*P*-value
	Before treatment	After treatment	
Preschool-aged children (3–5 years)	*n* = 319	*n* = 298	0.726^*^
*S. mansoni*^a^	66.5 (60.9–71.6)	19.1 (14.9–24.2)	< 0.001^*^
*S. mansoni*^b^			
Light	20.7 (16.4–25.6)	9.4 (6.4–13.4)	< 0.001^**^
Moderate	10.7 (7.6–14.7)	1.0 (0.3–3.2)	
Heavy	7.8 (5.2–11.5)	0.0	
*Trichuris trichiura*	1.3 (0.4–3.4)	0.0	1.000^**^
Hookworm	0.3 (0.0–2.0)	0.3 (0.0–2.2)	0.374^**^
Malaria (*P. falciparum*)	3.8 (2.1–6.7)	5.0 (2.9–8.3)	0.565^*^
FOB	41.7 (36.3–47.3)	29.2 (24.2–34.8)	< 0.01^*^
Anemia status^c^	48.0 (42.3–53.6)	38.6 (33.1–44.4)	0.023^*^
School-aged children (9–14 years)	*n* = 292	*n* = 286	0.726^*^
*S. mansoni*^a^	77.4 (72.1–82.0)	20.6 (16.2–25.9)	< 0.001^*^
*S. mansoni*^b^			
Light	19.9 (15.5–25.0)	8.4 (5.6–12.4)	< 0.001^**^
Moderate	23.6 (19.0–29.0)	1.7 (0.6–4.3)	
Heavy	24.0 (19.3–29.4)	1.0 (0.3–3.3)	
*Trichuris trichiura*	0.3 (0.0–2.2)	0.3 (0.0–2.2)	1.000^**^
Hookworm	0.0	0.3 (0.0–2.2)	0.495^**^
Malaria (*P. falciparum*)	5.1 (3.0–8.5)	8.4 (5.6–12.4)	0.163^*^
FOB	66.1 (60.3–71.4)	29.0 (23.9–34.7)	< 0.001^*^
Anemia^c^	34.2 (28.9–40.0)	32.9 (27.5–38.7)	0.793^*^

^a^Overall prevalence of *S. mansoni* infection based on both Kato–Katz and circulating cathodic antigen (CCA) tests

^b^Infection intensity defined by eggs per gram (epg) of stool: light (1–99 epg), moderate (100–399 epg), heavy (≥ 400 epg)

^c^Age-specific hemoglobin cut-offs for anemia: Hb < 110 g/L (< 5 years), Hb < 115 g/L (5–11 years), Hb < 120 g/L (12–14 years)

^*^P-values based on Pearson’s Chi-squared test

^**^P-values based on Fisher’s Exact test

**Fig. 2:**
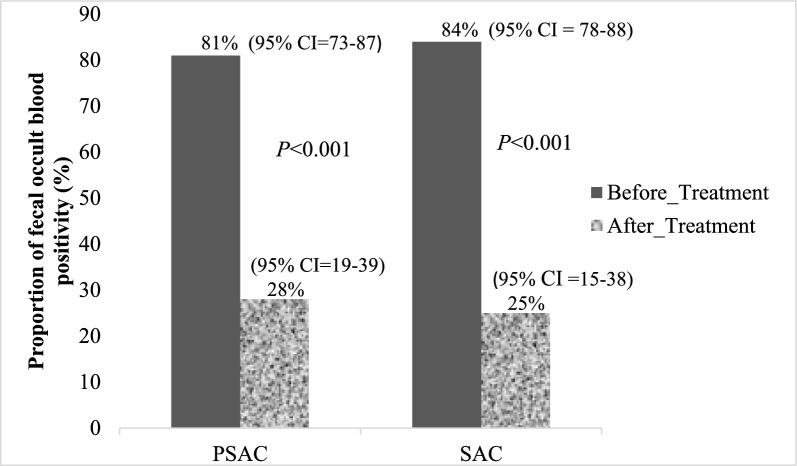
Proportion of fecal occult blood positivity among *S. mansoni*-infected preschool-aged and school-aged children before and after praziquantel treatment. *PSAC* preschool-aged children, *SAC* school-aged children

Among children infected with *S. mansoni*, fecal occult blood (FOB) positivity was high before treatment in both PSAC and SAC, at 81% (95% CI 73–87) and 84% (95% CI 78–88), respectively. Six weeks after praziquantel treatment, FOB positivity declined to 28% (95% CI 19–39) in PSAC and 25% (95% CI 15–38) in SAC. These reductions were statistically significant (*P* < 0.001 for both groups). The reduction in FOB positivity was observed following praziquantel administration, which may reflect a decrease in intestinal morbidity among infected children, as illustrated in Fig. 2.

Among children who tested negative for *S. mansoni* infection at baseline, FOB positivity was observed in a notable subset. Specifically, 22.4% (24/107) of preschool-aged children and 24.2% (16/66) of school-aged children were FOB positive before treatment. After treatment, FOB positivity among *S. mansoni* negative was 25.1% (61/241) and 21.1% (48/227) for preschool- and school-aged children, respectively (Table 3). These results indicate the presence of intestinal bleeding in children without detectable *S. mansoni* infection.

**Table 3:** Proportion of fecal occult blood positivity among *S. mansoni*-negative children before and after praziquantel treatment (*N* = 641)

Variable	FOB positive *n*(%) (95% CL)	FOB negative* n*(%) (95% CL)	*P*-value (Pearson's Chi-square)
Preschool-aged children (3–5 years)			
Before treatment	24 (22.4) (14.9–32.3)	83 (77.6) (67.7–85.1)	0.658
After treatment	61 (25.1) (19.9–31.1)	180 (74.6) (68.9–80.0)	
School-aged children (9–14 years)			
Before treatment	16 (24.2) (15.0–36.5)	50 (75.8) (63.5–85.0)	0.713
After treatment	48 (21.1) (16.0–27.3)	179 (78.9) (72.7–84.0)	

School-aged children who tested positive for *S. mansoni* infection had approximately ten times higher odds of fecal occult blood positivity (*P* < 0.001). Preschool-aged children infected with *S. mansoni* were about three times more likely to exhibit positive FOB compared to uninfected children (*P* < 0.01), as shown in Table 4. Additionally, PSAC residing on the Island showed a two-fold higher likelihood of FOB positivity compared to those from mainland locations, although this was statistically significant only before treatment (*P* = 0.01). Female PSAC with *S. mansoni* infection had 1.5 times higher odds of FOB positivity; however, this association was not statistically significant (*P* = 0.085). A significant association between *S. mansoni* infection and FOB was evident in both PSAC and SAC after adjusting for gender and location (*P* < 0.01). Low hemoglobin levels indicative of anemia did not show a significant association with FOB positivity in either group when considering both malaria and *S. mansoni* infection status (Table 4).

**Table 4:** Association between *S. mansoni* infection and fecal occult blood (FOB) among children before and after praziquantel treatment

Variable		Fecal occult blood (FOB)			
		Before treatment		After treatment	
		AOR (95% CI)	*P*	AOR (95% CI)	*P*
Preschool-aged children					
*S. mansoni*	Positive	2.5 (1.4–4.7)	0.002	2.5 (1.3–4.6)	0.005
Gender	Female	1.5 (0.9–2.5)	0.085	0.9 (0.5–1.5)	0.652
Location	Island	2.0 (1.2–3.4)	0.01	1.1 (0.6–1.9)	0.758
Anemia status	Anemic	1.0 (0.6–1.6)	0.975	1.3 (0.8–2.2)	0.342
Malaria	Positive	0.5 (0.1–1.9)	0.353	0.3 (0.1–1.3)	0.166
School-aged children					
*S. mansoni*	Positive	9.9 (5.0–20.6)	< 0.001	5.4 (2.9–10.6)	< 0.0001
Gender	Female	0.7 (0.4–1.3)	0.307	0.7 (0.4–1.3)	0.290
Location	Island	1.4 (0.7–2.8)	0.297	1.0 (0.5–1.8)	0.991
Anemia status	Anemic	1.0 (0.5–1.8)	0.899	0.8 (0.5–1.5)	0.586
Malaria	Positive	0.4 (0.1–1.5)	0.174	0.9 (0.3–2.4)	0.838

The infection status of *S. mansoni* was determined by both Kato–Katz (KK) and circulating cathodic antigen (CCA) tests

*FOB* fecal occult blood, *AOR* adjusted odds ratio, *CI* confidence interval

Covariates in the model: gender, location (mainland vs. island), anemia status, and malaria status

Reference groups: *S. mansoni*-negative, male, mainland, non-anemic, and malaria-negative

## Discussion

This pre–post study investigated whether the fecal occult blood (FOB) test could serve as a surrogate marker for assessing intestinal morbidities associated with *Schistosoma mansoni* infection before and after praziquantel treatment. The findings revealed a statistically significant association between *S. mansoni* infection and FOB positivity in both preschool- and school-aged children. Moreover, the observed decrease in FOB prevalence after treatment among infected children may reflect reduced intestinal morbidity, suggesting that FOB could be explored as a supplementary monitoring tool in schistosomiasis-endemic regions.

In our study area, 77.4% of school-aged children (SAC) and 66.5% of preschool-aged children (PSAC) were infected with *S. mansoni*. Most infected SAC harbored moderate to heavy infections, while PSAC typically had light infection intensities. These findings are consistent with characteristic age-prevalence and age-intensity patterns of schistosomiasis, where infections often begin as early as 2 years of age and peak between 10 and 14 years [6]. The higher prevalence and intensity among SAC likely reflect their increased exposure to infested water through activities such as swimming [28].

The persistently high transmission in this region [26, 29, 32], despite mass drug administration (MDA) campaigns initiated over a decade ago, may be influenced by a combination of environmental, behavioral, and infrastructural factors, including limited access to safe water and sanitation, frequent contact with natural water bodies, and the continued presence of intermediate host snails [33–35]. Additionally, annual MDA for intestinal schistosomiasis had not been implemented in the study area prior to this investigation, which may partly explain the high infection levels observed at baseline.

Before treatment, FOB positivity was recorded in 66.1% of SAC and 41.7% of PSAC, underscoring the presence of intestinal morbidity even among younger age groups. These findings support growing evidence that preschool-aged children are not only at risk of *S. mansoni* infection but may also experience associated morbidity[10]. Additionally, FOB positivity rates were significantly higher among children infected with *S. mansoni* compared to those who were uninfected, suggesting a possible association between infection and gastrointestinal bleeding detectable by FOB tests. Such bleeding may result from intestinal mucosal damage caused by schistosome egg deposition and subsequent inflammation [6, 36]. Six weeks after treatment, the prevalence of FOB among infected schoolchildren decreased significantly, corresponding with the reduction in infection intensity. This decline may indicate a treatment-related effect but could also reflect temporary improvements in the intestinal mucosa.

We observed a positive correlation between *S. mansoni* infection and FOB positivity in this study population. This finding aligns with previous studies [23, 24, 37] that have highlighted the feasibility of FOB as a field-appropriate tool for monitoring changes in intestinal morbidity following chemotherapy. Our study extends these findings by focusing on an older cohort of schoolchildren aged 9–14 years, a group underrepresented in earlier research. The use of a two-dose regimen of praziquantel, administered 2 weeks apart, was intended to enhance treatment efficacy in an area where frequent water contact increases the likelihood of reinfection. However, findings from Egypt [21] did not demonstrate a significant association between FOB and *S. mansoni*, which may reflect differences in infection intensity. While heavier worm burdens are generally associated with more severe schistosomiasis complications, the relationship is not fully understood [14, 38].

In the context of *S. japonicum*, heavy infections and occult blood loss have been implicated in anemia [19], but mechanisms remain unclear, particularly in malaria-endemic regions such as western Kenya [8, 30]. In our study, after adjusting for malaria, evidence linking FOB to anemia was insufficient, suggesting that other factors contribute to anemia in the region. Past research has also indicated that, in addition to schistosomiasis, soil-transmitted helminths (STH) such as *Trichuris trichiura* and hookworm can cause intestinal bleeding [39, 40]. However, in the context of FOB, the correlation with STH remains uncertain due to insufficient evidence [20, 22, 41]. In our study, the overall prevalence of *T. trichiura* and hookworm was low (< 2%), likely due to annual deworming programs in the area [13, 42]. This finding is consistent with previous studies reporting low prevalence of STH in western Kenya [26, 27].

In schistosomiasis-endemic areas, the effectiveness of mass treatment with praziquantel is typically monitored using infection prevalence and intensity as proxy indicators of morbidity [12, 13]. While these indicators are widely used, they may not fully reflect the extent of disease burden, particularly in young children. The relationship between infection intensity and morbidity is inconsistent, especially in cases of *S. mansoni* [43]. In this study, we observed a significant association between *S. mansoni* infection and fecal occult blood (FOB) positivity among preschool- and school-aged children. This suggests that FOB testing, by detecting signs of intestinal morbidity, could serve as a complementary tool for monitoring schistosomiasis-related morbidity, especially in young populations where routine infection parameters may underestimate the true burden.

### Limitations of the study

While a significant reduction in fecal occult blood (FOB) positivity was observed following praziquantel treatment among *S. mansoni*-infected children, the FOB assay used in this study was qualitative and did not allow for assessing the severity of intestinal bleeding. This limited the ability to explore associations between the intensity of infection and the degree of morbidity. In addition, FOB positivity was detected in a notable proportion of children who tested negative for *S. mansoni*, with 22.4% of preschool-aged and 24.2% of school-aged children showing positive results. This suggests that other factors may contribute to background intestinal bleeding. Common enteric pathogens such as *Shigella*, enterohemorrhagic *Escherichia coli*, *Giardia lamblia*, and *Entamoeba histolytica*, which were not assessed in this study, may also play a role. Future studies incorporating quantitative FOB methods and broader diagnostics will be valuable for validating the role of FOB in routine schistosomiasis morbidity monitoring.

## Conclusions

This study demonstrates a significant association between *S. mansoni* infection and fecal occult blood positivity among preschool- and school-aged children in a high-endemicity setting. The significant post-treatment reductions in both infection prevalence and FOB positivity suggest the potential utility of FOB testing as a non-invasive, field-applicable tool for monitoring intestinal morbidity related to schistosomiasis. While background FOB positivity among uninfected individuals calls for cautious interpretation, these findings contribute to growing evidence that practical morbidity indicators such as FOB could support national and global schistosomiasis control and elimination strategies, particularly in resource-limited endemic areas.

## Data Availability

All data analyzed during this study are included in this published article. The datasets used during analysis are available from the corresponding author on reasonable request.

## Abbreviations

CCA: Circulating cathodic antigen
DALYs: Disability Adjusted Life Years
EPG: Egg counts per gram
HDSS: Health and demographic surveillance system
KEMRI: Kenya Medical Research Institute
MDA: Mass drug administration
NUITM: Nagasaki University, Institute of Tropical Medicine
POC: Point-of-care
PSAC: Preschool-aged children
PP1: Preprimary one
PZQ: Praziquantel
SAC: School-aged children
STH: Soil-transmitted helminths
WHO: World Health Organization
